# Green Tea, Coffee, and Caffeine Consumption Are Inversely Associated with Self-Report Lifetime Depression in the Korean Population

**DOI:** 10.3390/nu10091201

**Published:** 2018-09-01

**Authors:** Jiwon Kim, Jihye Kim

**Affiliations:** Department of Medical Nutrition, Graduate School of East-West Medical Science, Kyung Hee University, Yongin 17104, Korea; jiwonns@khu.ac.kr

**Keywords:** green tea, coffee, caffeine, depression

## Abstract

This study investigated the associations of green tea, coffee, and caffeine consumption with self-report lifetime depression in the Korean population using data from the Korean National Health and Nutrition Examination Survey. In total, 9576 participants (3852 men and 5724 women) aged 19 years or older were selected for the present study. Green tea, coffee, and caffeine consumption levels were assessed with a validated food frequency questionnaire. Multivariate logistic regression analysis was used to determine the odds ratios (OR) and 95% confidence intervals (CIs) for depression according to green tea, coffee, and caffeine consumption. Frequent green tea consumers (≥3 cups/week) had 21% lower prevalence of depression (OR = 0.79, 95% CI = 0.63–0.99, *p* for trend = 0.0101) than green tea non-consumers after adjustment for potential confounders. Likewise, frequent coffee drinkers (≥2 cups/day) had 32% lower prevalence of depression (OR = 0.68, 95% CI = 0.55–0.85, *p* for trend = 0.0026) than coffee non-drinkers after adjustment for potential confounders. Also, participants in the highest quartile of caffeine consumption had 24% lower prevalence of depression than those in the lowest quartile (OR = 0.76, 95% CI = 0.62–0.92, *p* for trend = 0.0032). Frequent consumption of green tea, coffee, or caffeine was associated with a reduced prevalence of self-report lifetime depression in Korean adults. A prospective study and randomized clinical trials should be conducted to confirm the inverse relationships of green tea and coffee consumption with risk of depression.

## 1. Introduction

Depression is one of the most common diseases worldwide and has a heavy socioeconomic burden [1,2]. Studies from different countries worldwide have indicated that more than 90% of suicide victims or attempters in the general population had at least one major depressive symptom [2,3]. Depression has been ranked third on the World Health Organization’s list of medical conditions with the greatest disease burden worldwide and is expected to top the list by 2030 [4]. According to 2017 health statistics from the Organization for Economic Cooperation and Development (OECD), the suicide rate of Koreans is steadily increasing and is the second highest among OECD countries [5].

Epidemiological studies have demonstrated that green tea and coffee consumption are inversely associated with depression. However, some studies have had conflicting results. Greater consumption of green tea was inversely associated with depressive symptoms in a Japanese population [6,7], whereas no association was reported in middle-aged Finnish men [8]. Inverse association between coffee intake and depression was observed in a US population and Finnish population [8,9], whereas another study found no such association in the Finnish general population [10]. Caffeine, a major component of green tea and coffee, may also be associated with depression. In the UK, greater consumption of caffeine was associated with a higher risk of depression and anxiety [11]. 

Green tea and coffee are two of the most consumed beverages worldwide, and their health effects have caused wide interest. Green tea, which accounts for 20% of the total tea consumption worldwide, is the primary beverage consumed in Asian countries [12]. In Japan, 53% of adults consume green tea on a daily basis [6], and in China, 46.1% of adults regularly drink tea [13]. In Korea, green tea consumption was 2.9 g/tea drinker/day in 2016 [14]. Recent data have indicated that coffee intake is rapidly increasing in the Korean population. Koreans drink coffee approximately 12 times per week, and coffee ranks the highest among the top five most frequently consumed foods or beverages [15]. 

However, the relationships of green tea and coffee consumption with the risk of depression in the Korean population have not been investigated. Therefore, we examined the associations of green tea, coffee, and caffeine consumption with the risk of self-report lifetime depression in the general Korean population using nationally representative survey data.

## 2. Materials and Methods 

### 2.1. Study Population

This study was based on the fifth Korean National Health and Nutrition Examination Survey (KNHANES), a nationally representative survey carried out by the Korea Centers for Disease Control and Prevention (KCDC) in 2010, 2011, and 2012. The survey employed a stratified, multistage, probability-sampling design to represent the entire Korean population. Of the 25,534 Koreans who participated in the fifth KNHANES, 19,599 aged 19 years or older were selected for the present study. The following individuals were excluded: 1533 who did not complete the depression questions; 7571 who did not complete the food frequency questionnaire (FFQ); 243 with insufficient information on socioeconomic and anthropometric measurements; and 676 who had cardiovascular disease or cancer. Thus, in total, 9576 Korean adults (3852 men and 5724 women) were eligible for the analysis. Written informed consent was obtained from all study participants. The study was approved by the KCDC Institutional Review Board. 

### 2.2. Dietary Assessment

Dietary intake was evaluated with a validated FFQ [16] through face-to-face interviews with trained dietitians. The average from two FFQs conducted in 2010 and 2011 was used to estimate green tea and coffee consumption. Green tea and coffee consumption levels were estimated with the question “How often did you drink green tea (or coffee) during the past year?” on the FFQ. The responses included 10 options (almost never, 6–11 times/year, once a month, 2–3 times/month, once a week, 2–3 times/week, 4–6 times/week, once a day, 2 times/day, and 3 times/day). Green tea consumption was categorized into four groups (never, 0–≤1, 1–<3, and ≥3 cups/week). Coffee consumption was categorized into four groups (never, 0–<1, 1–<2, and ≥2 cups/day). The daily intake of individual foods was determined, and the total intake of each food was summed. Twelve vegetable items; 11 fruit items; four meats, including beef, pork, chicken, and processed meat (ham, sausage, etc.); and nine fish items (mackerel, hairtail, anchovy, etc.) were assessed. For calculation of energy intake, dietary intake was determined by the 24-h recall method. Energy intake was estimated from the food composition table of the Rural Development Administration [17]. Daily caffeine consumption was estimated with the assumption that 100 mL of green tea and coffee contained 20 mg and 60 mg of caffeine, respectively [6]. 

### 2.3. Definition of Depression

Depression was assessed by the following questions: “Have you ever been diagnosed with depression by a physician?” (*n* = 371) and “Do you have depression at present?” (*n* = 171) and “Are you getting treatment for depression at present?” (*n* = 108) and “In the past year, have you felt sadness or despair continuously for two or more weeks that was severe enough to interfere with daily life?” (*n* = 1221). Participants who reported at least one “yes” were regarded as having depression. 

### 2.4. Covariates

Data on demographic characteristics, socioeconomic status, and lifestyle factors were obtained with a self-administered questionnaire and verified in a personal interview. The monthly income level was classified as low, medium, or high. The education level was classified as ≤6 years (elementary school graduates), 7 to ≤12 years (middle or high school graduates), or >12 years (college graduate or more). Participants were classified into the following three groups according to smoking status: lifetime non-smoker, former smoker (for at least 1 year), and current smoker. The frequency of alcohol use during the previous year was assessed by a questionnaire, and responses were converted into alcohol intake per week. Then, participants were categorized into three groups: lifetime non-drinker, light or moderate drinker (<2 times/week), and heavy drinker (≥2 times/week). Regarding physical activity, participants were classified based on the amount of regular exercise performed, as follows: vigorous exercise for ≥20 min/time and ≥5 times/week; moderate exercise for ≥30 min/time and ≥5 times/week; or walking for ≥30 min/time and ≥5 times/week. Height was measured to the nearest 0.1 cm with a stadiometer (Seca-225, Seca Corporation, Hamburg, Germany), and body weight was measured to the nearest 0.1 kg with a body weight scale (GL-6000-20, G-tech Corporation, Uijeongbu city, Korea) while subjects wore light clothes without shoes. Body mass index (BMI) was calculated as weight (kg) divided by height squared (m^2^). 

### 2.5. Statistical Analysis

All data analyses were performed with SAS software version 9.4 (SAS Institute, Cary, NC, USA) [18]. Sample weights were applied in all analyses so that the data would reflect national population estimates. Data are expressed as numbers and percentages (categorical) or as means ± standard errors (SEs). The Chi-square test was used to compare proportions across groups of categorical variables by the PROC SURVEYFREQ procedure. Mean values and standard errors of continuous variables were calculated by the PROC SURVEYMEANS procedure, and the PROC SURVEYREG procedure was used to compare the differences of variables according to the categories of green tea or coffee consumption. A multivariable-adjusted logistic regression analysis was conducted to determine the odds ratios (ORs) and 95% confidence intervals (CIs) for depression according to green tea, coffee, and caffeine consumption by means of the PROC SURVEYLOGISTIC procedure. In the multivariate adjusted models, model 1 was adjusted for age and sex; model 2 was adjusted for the covariates in model 1 plus education level, income level, smoking status, alcohol intake, physical activity, and BMI; and model 3 was adjusted for the covariates in model 2 plus the intakes of energy, vegetables, fruits, meat, fish, and green tea or coffee. *p*-values < 0.05 were considered to be statistically significant.

## 3. Results

### 3.1. Characteristics by Presence of Depression

The characteristics of participants according to the presence of depression are presented in Table 1. Among the 9576 participants, 15.1% indicated that they had suffered from depression or depressive symptoms during their lives. Participants who had depression were older, more likely to be women, to have a low income, less likely to be educated, to be lifetime non-smokers, to be lifetime non-drinkers, and to be regular exercisers. Also, participants who had depression had lower intakes of total energy, vegetables, fruits, meat, fish, green tea, and coffee than those who did not have depression. 

Baseline characteristics of 9576 participants who were included in this analysis, and 10,023 participants who were excluded from the analysis were compared (Supplementary File Table S1). Participants who were included from the analysis were younger, more likely to be women, to be never smokers, to be regular exercisers compared with those who were excluded in the analysis. Participants who were included had higher intakes of total energy, coffee, and red meat compared to those who were excluded in the analysis. 

### 3.2. Characteristics by Green Tea Consumption

The characteristics of participants according to consumption of green tea are presented in Table 2. Frequent green tea consumers (≥3 cups/week) were younger, more likely to be men, to have a high income, and to be educated, and less likely to be lifetime non-smokers and current alcohol drinkers than green tea non-consumers (never). Frequent green tea consumers were more likely to perform physical activity regularly and to have higher BMI than green tea non-consumers. Also, frequent green tea consumers had higher intakes of total energy, vegetables, fruits, meat, fish, and coffee than infrequent green tea consumers.

### 3.3. Characteristics by Coffee Consumption

The characteristics of participants according to consumption of coffee are presented in Table 3. Frequent coffee drinkers (≥2 cups/day) were older, more likely to be men, to have a high income, and to be educated, and less likely to be lifetime non-smokers and current alcohol drinkers than coffee non-drinkers (never). Frequent coffee drinkers had higher BMI and higher intakes of total energy, vegetables, fish, and green tea than infrequent coffee drinkers.

### 3.4. Association between Green Tea, Coffee and Caffeine Consumption and the Prevalence of Depression

The adjusted odds ratios and 95% confidence intervals for depression according to frequency of green tea, coffee and caffeine consumption are presented in Table 4. The prevalence of depression decreased as green tea and coffee consumption increased. Frequent green tea consumers (≥3 cups/week) had 21% lower prevalence of depression (OR = 0.79, 95% CI = 0.63–0.99, *p* for trend = 0.01) than green tea non-consumers after adjustment for the potential confounders of age, sex, BMI, income level, education level, alcohol intake, smoking status, physical activity, and the intakes of energy, vegetables, fruits, meat, fish, and coffee. Likewise, frequent coffee drinkers (≥2 cups/day) had 32% lower prevalence of depression (OR = 0.68, 95% CI = 0.55–0.85, *p* for trend = 0.003) than coffee non-drinkers after adjustment for potential confounders. The prevalence of depression decreased as caffeine consumption increased. Participants in the highest quartile of caffeine consumption had 24% lower prevalence of depression than those in the lowest quartile after adjustment for potential confounders (OR = 0.76, 95% CI = 0.62–0.92, *p* for trend = 0.003). In sensitivity analysis, an inverse association of green tea, coffee, and caffeine consumption with depression risk remained.

## 4. Discussion

This study revealed that green tea and coffee consumption were inversely associated with self-report lifetime depression in Korean adults, based on data from a nationally representative survey. The prevalence of depression in frequent green tea consumers was 21% lower than in green tea non-consumers after adjustment for potential confounders. The prevalence of depression in frequent coffee drinkers was 32% lower than in coffee non-drinkers after adjustment for potential confounders. In addition, the prevalence of depression was 24% lower in participants in the highest quartile of caffeine consumption from green tea and coffee than in those in the lowest quartile. These results suggest that frequent green tea and coffee consumption may help in the prevention of depression. 

Similar to these findings, previous studies have supported the inverse relationships of green tea, coffee, and caffeine consumption with depression. In a community-based study, daily tea consumption of 1 cup/day was associated with a 41% lower risk of depressive symptoms than non or irregular tea consumption group in an older Chinese population [13]. Among 1,058 Japanese older people, more frequent consumption of green tea (≥4 cups/day) was associated with a 52% lower prevalence of severe depressive symptoms than infrequent green tea consumption (≤1 cup/day) [19]. Higher coffee intake (≥2 cups/day) was associated with a 39% lower prevalence of depressive symptom than lower coffee intake (<1 cup/day) in Japanese men and women aged 20–68 years [6].

In a 10-year follow-up study of women in the US, individuals who frequently drank caffeinated coffee (≥4 cups/day) had a 20% lower risk of incident depression than those who infrequently drank caffeinated coffee (≤1 cup/week) after adjustment for potential risk factors, whereas decaffeinated coffee consumption was not associated with depression risk [20]. This prospective study also indicated that higher caffeine consumption (≥550 mg/day) was associated with a 20% lower risk of depression than lower caffeine consumption (<100 mg/day).

Several mechanisms have been suggested to explain the negative associations of green tea and coffee consumption with depression risk. Green tea is rich in polyphenols, mainly catechins [21]. Oral administration of green tea polyphenol for 7 days significantly reduced immobility in a mouse model of depression, suggesting that this polyphenol has antidepressant-like effects [22]. Green tea polyphenols also reduced corticosterone and adrenocorticotropic hormone levels, thus reducing maladaptive responses to stress by inhibiting the hypothalamic–pituitary–adrenal axis [23]. Stress is well known to be one of the most important factors responsible for depressive disorders. 

Theanine, an amino acid found in green tea [21], has recently been reported to have neuroprotective effects by modulating neurotransmitters and to reduce psychological stress. An animal study demonstrated that theanine has an antidepressant effect [24]. Theanine may regulate the concentrations of several brain neurotransmitters, including dopamine and serotonin [25], dysfunction of which is a possible cause of depression [26]. Coffee is also rich in biologically active substances such as chlorogenic acid, nicotinic acid, trigonelline, quinolinic acid, tannic acid, and pyrogallic acid [27,28]. Chlorogenic acid and caffeic acid have been found to have anti-inflammatory and antioxidant effects in vivo [29,30] and in vitro [31]. Inflammation and oxidation may contribute to the pathophysiology of depression [32,33,34]. Caffeine is well known to have psychostimulant effects [35]. Caffeine modulates dopaminergic neurotransmission as a non-selective adenosine receptor antagonist in the brain [35,36,37]. The antagonistic effect of caffeine on adenosine may also imply that caffeine acts through non-dopaminergic mechanisms; for instance, by modulating the release of acetylcholine and serotonin [35].

The present study had several limitations. First, it was not possible to identify causal relationships of green tea, coffee, and caffeine consumption with the risk of depression, because this study was designed cross-sectionally. Second, we did not investigate the impact of various types of coffee and tea on depression. Third, most participants were diagnosed with depression by self-report, not through semi-structured assessment tools and, thus, there might be the potential misjudgment for depression.

Despite these limitations, to the best of our knowledge, this was the first study to investigate the relationships of green tea and coffee consumption with depression risk in the general Korean population. The strength of our study was the use of data from the largest nationally representative survey in the general Korean population. 

## 5. Conclusions

In conclusion, frequent green tea (≥3 cups/week) and coffee consumption (≥2 cups/day) were inversely associated with self-report lifetime depression after adjustment for potential confounders in Korean adults. Also, higher consumption of caffeine from green tea and coffee was associated with a lower prevalence of depression. These results suggest that increasing green tea and coffee consumption may help to prevent and manage depression in Korean adults. In future research, high-quality randomized controlled trials should be conducted to verify these associations.

## Figures and Tables

**Table 1:** Characteristics of participants by depression status (*n* = 9576) ^a^.

	Depression (*n* = 1443)	Non-Depression (*n* = 8133)	*p* Value
Age (year)	46.2 ± 0.5	43.9 ± 0.3	<0.0001
No. of participants (%)			<0.0001
Men	356 (33.6)	3496 (52.2)	
Women	1087 (66.4)	4637 (47.8)	
Income level (%)			0.0001
Low	433 (31.8)	1872 (25.2)	
Medium	707 (48.9)	4189 (51.5)	
High	303 (19.4)	2072 (23.2)	
Educational level (%)			<0.0001
Elementary school (≤6 years)	509 (27.4)	1883 (15.8)	
Middle/high school (7–12 years)	620 (47.9)	3610 (48.3)	
College or higher (>12 years)	314 (24.7)	2640 (35.9)	
Smoking status (%)			0.03
Never	960 (57.5)	4850 (53.0)	
Former	225 (17.2)	1676 (20.5)	
Current	258 (25.3)	1607 (26.5)	
Alcohol consumption (%)			0.002
Never	465 (25.7)	2119 (20.5)	
<2 times/week	725 (53.4)	4306 (56.1)	
≥2 times/week	253 (20.9)	1708 (23.4)	
Physical activity			
Regular	657 (46.5)	3861 (49.1)	0.12
Body mass index (BMI) (kg/m²)	23.6 ± 0.1	23.6 ± 0.1	0.61
Energy intake (kcal/day)	1963.7 ± 31.6	2139.3 ± 16.6	<0.0001
Beverage intake			
Green tea (cups/week)	1.50 ± 0.11	1.91 ± 0.06	0.001
Coffee (cups/day)	1.25 ± 0.03	1.37 ± 0.02	0.001
Food intake (times/day)			
Vegetable	4.10 ± 0.06	4.22 ± 0.03	0.06
Fruit	0.98 ± 0.02	1.04 ± 0.01	0.02
Red meat	0.47 ± 0.02	0.54 ± 0.01	0.0001
Fish	0.88 ± 0.03	0.94 ± 0.01	0.03

^a^ Values are means ± standard error (SE) or numbers (percentages); All *p* values are significant at *p* < 0.05.

**Table 2:** Demographic characteristics of subjects according to green tea consumption (*n* = 9576) ^a^.

	Never	0–≤1 Cup/Week	1–<3 Cups/Week	≥3 Cups/Week	*p* Value
Participants No. (No. of cases)	3828 (682)	3455 (498)	967 (110)	1326 (153)	<0.0001
Sex (%) Men	1477 (46.3)	1375 (50.4)	383 (50.7)	617 (54.2)	0.0004
Women	2351 (53.7)	2080 (49.6)	584 (49.3)	709 (45.8)	
Age (year)	48.0 ± 0.5	41.9 ± 0.4	41.1 ± 0.5	43.0 ± 0.5	<0.0001
Income level (%)					<0.0001
Low	1144 (31.4)	729 (23.8)	199 (23.2)	233 (20.9)	
Medium	1929 (50.8)	1795 (51.1)	500 (53.2)	672 (50.6)	
High	755 (17.8)	931 (25.0)	268 (23.6)	421 (28.6)	
Educational level (%)					<0.0001
Elementary school (≤6 years)	1463 (28.3)	650 (12.9)	113 (7.4)	166 (8.8)	
Middle/high school (7–12 years)	1578 (48.2)	1626 (49.6)	452 (48.1)	574 (44.6)	
College or higher (>12 years)	787 (23.5)	1179 (37.5)	402 (44.5)	586 (46.5)	
Smoking status (%)					0.045
Never	23,901 (53.5)	2167 (55.6)	608 (53.6)	734 (48.9)	
Former	754 (19.5)	666 (19.6)	183 (20.5)	298 (22.0)	
Current	773 (27.0)	622 (24.8)	176 (25.9)	294 (29.1)	
Alcohol consumption (%)					<0.0001
Never	1291 (26.6)	848 (19.5)	185 (15.1)	260 (16.1)	
<2 times/week	1748 (51.3)	1942 (58.0)	586 (60.6)	755 (57.7)	
≥2 times/week	789 (22.1)	665 (22.4)	196 (24.2)	311 (26.2)	
Physical activity					
Regular	1679 (45.1)	1670 (50.1)	478 (51.0)	691 (52.7)	0.001
Body mass index (kg/m²)	23.5 ± 0.1	23.6 ± 0.1	23.7 ± 0.1	23.9 ± 0.1	0.01
Energy intake (kcal/day)	2021.3 ± 23.9	2151.4 ± 21.8	2150.9 ± 38.7	2230.5 ± 37.0	<0.0001
Beverage intake					
Coffee (times/day)	1.36 ± 0.02	1.27 ± 0.02	1.28 ± 0.04	1.59 ± 0.04	<0.0001
Food intake (times/day)					
Vegetable	3.93 ± 0.04	4.12 ± 0.04	4.61 ± 0.08	4.82 ± 0.07	<0.0001
Fruit	0.89 ± 0.02	1.06 ± 0.02	1.18 ± 0.03	1.25 ± 0.03	<0.0001
Red meat	0.44 ± 0.01	0.55 ± 0.01	0.64 ± 0.02	0.61 ± 0.02	<0.0001
Fish	0.81 ± 0.01	0.91 ± 0.01	1.11 ± 0.03	1.15 ± 0.03	<0.0001

^a^ Values are means ± SE or numbers (percentages). All *p* values are significant at *p* < 0.05.

**Table 3:** Demographic characteristics of subjects according to coffee consumption (*n* = 9576) ^a^.

	Never	0–<1 Cup/Day	1–<2 Cups/Day	≥2 Cups/Day	*p* Value
Participants No. (No. of cases)	964 (196)	2301 (372)	2330 (355)	3981 (520)	<0.0001
Sex (%) Men	296 (40.2)	823 (47.0)	770 (41.7)	1963 (57.2)	<0.0001
Women	668 (59.8)	1478 (53.0)	1560 (58.3)	2018 (42.8)	
Age (year)	43.7 ± 0.8	41.0 ± 0.5	46.4 ± 0.5	45.2 ± 0.3	<0.0001
Income level (%)					0.001
Low	288 (32.8)	586 (27.7)	567 (25.9)	864 (23.9)	
Medium	467 (46.7)	1173 (51.3)	1177 (50.0)	2079 (52.7)	
High	209 (20.5)	542 (21.0)	586 (24.1)	1038 (23.4)	
Educational level (%)					<0.0001
Elementary school (≤6 years)	365 (24.9)	617 (17.5)	672 (20.9)	738 (14.0)	
Middle/high school (7–12 years)	350 (42.8)	1000 (50.0)	983 (45.3)	1897 (50.0)	
College or higher (>12 years)	249 (32.3)	684 (32.5)	675 (33.8)	1346 (36.1)	
Smoking status (%)					<0.0001
Never	701 (65.9)	1540 (59.3)	1553 (60.0)	2016 (44.1)	
Former	159 (17.1)	441 (19.5)	442 (19.4)	859 (21.3)	
Current	104 (17.0)	320 (21.2)	335 (20.6)	1106 (34.6)	
Alcohol consumption (%)					<0.0001
Never	441 (34.7)	673 (21.6)	668 (24.2)	802 (16.4)	
<2 times/week	380 (47.4)	1236 (59.3)	1236 (55.1)	2179 (55.8)	
≥2 times/week	143 (17.9)	392 (19.1)	426 (20.7)	1000 (27.8)	
Physical activity					0.48
Regular	432 (46.9)	1122 (50.3)	1097 (48.6)	1867 (48.3)	
Body mass index (kg/m²)	23.2 ± 0.2	23.2 ± 0.1	23.7 ± 0.1	23.9 ± 0.1	<0.0001
Energy intake (kcal/day)	1971.4 ± 38.1	2077.7 ± 27.1	1969.0 ± 24.9	2244.9 ± 21.3	<0.0001
Beverage intake					
Green tea (times/week)	1.17 ± 0.14	1.33 ± 0.08	1.97 ± 0.10	2.25 ± 0.10	<0.0001
Food intake (times/day)					
Vegetable	4.12 ± 0.10	3.92 ± 0.05	4.27 ± 0.05	4.35 ± 0.04	<0.0001
Fruit	1.02 ± 0.04	1.03 ± 0.02	1.08 ± 0.02	1.01 ± 0.02	0.08
Red meat	0.53 ± 0.02	0.54 ± 0.01	0.51 ± 0.02	0.54 ± 0.01	0.49
Fish	0.89 ± 0.03	0.86 ± 0.02	0.92 ± 0.02	0.98 ± 0.01	<0.0001

^a^ Values are means ± SE or numbers (percentages); All *p* values are significant at *p* < 0.05.

**Table 4:** Odds ratios (ORs) and 95% confidence intervals (CIs) of depression according to green tea, coffee and caffeine consumption (*n* = 9576).

Green Tea	Never	0–≤1 Cup/Week	1–<3 Cups/Week	≥3 Cups/Week	*p* for Trend
Participants No. (No. of cases)	3828 (682)	3455 (498)	967 (110)	1326 (153)	
Unadjusted	1.00	0.73 (0.62–0.86)	0.53 (0.41–0.68)	0.62 (0.50–0.77)	<0.0001
Model 1 ^∫^	1.00	0.78 (0.66–0.92)	0.57 (0.44–0.73)	0.67 (0.54–0.84)	<0.0001
Model 2 ^∬^	1.00	0.85 (0.72–1.01)	0.64 (0.49–0.83)	0.76 (0.61–0.96)	0.004
Model 3 ^∮^	1.00	0.85 (0.72–1.01)	0.65 (0.50–0.85)	0.79 (0.63–0.99)	0.01
Sensitivity analysis ^§^	3828 (617)	3455 (443)	967 (102)	1326 (132)	
	1.00	0.87 (0.73–1.03)	0.69 (0.52–0.91)	0.75 (0.58–0.98)	0.03
Coffee	Never	0–<1 cup/day	1–<2 cups/day	≥2 cups/day	*p* for trend
Participants No. (No. of cases)	964 (196)	2301 (372)	2330 (355)	3981 (520)	
Unadjusted	1.00	0.68 (0.53–0.87)	0.62 (0.49–0.79)	0.60 (0.49–0.74)	<0.0001
Model 1 ^∫^	1.00	0.73 (0.57–0.93)	0.61 (0.48–0.78)	0.67 (0.54-0.83)	0.001
Model 2 ^∬^	1.00	0.73 (0.57–0.93)	0.63 (0.49–0.80)	0.37 (0.54–0.83)	0.001
Model 3 ^∮^	1.00	0.74 (0.58–0.95)	0.65 (0.51–0.82)	0.68 (0.55–0.85)	0.003
Sensitivity analysis ^§^	964 (176)	2301 (328)	2330 (319)	3981 (471)	
	1.00	0.73 (0.56–0.95)	0.66 (0.52–0.85)	0.70 (0.56–0.95)	0.006
Caffeine	Q1 (≤22 mg/day)	Q2 (22–≤62 mg/day)	Q3 (62–≤ 122.9 mg/day)	Q4 (>122.9 mg/day)	*p* for trend
Participants No. (No. of cases)	2357 (430)	2382 (383)	2444 (345)	2393 (285)	
Unadjusted	1.00	0.75 (0.61–0.91)	0.70 (0.58–0.85)	0.66 (0.55–0.80)	<0.0001
Model 1 ^∫^	1.00	0.73 (0.60–0.90)	0.71 (0.59–0.85)	0.76 (0.63–0.92)	0.001
Model 2 ^∬^	1.00	0.74 (0.60–0.91)	0.73 (0.61–0.89)	0.75 (0.62–0.92)	0.002
Model 3 ^∮^	1.00	0.74 (0.61–0.91)	0.74 (0.61–0.89)	0.76 (0.62–0.92)	0.003
Sensitivity analysis ^§^	2357 (381)	2382 (344)	2444 (307)	2393 (262)	
	1.00	0.77 (0.63–0.94)	0.75 (0.62–0.91)	0.80 (0.65–0.97)	0.009

^∫^ Adjusted for age, and sex; ^∬^ Model 1 + BMI, income level, education level, alcohol intake, smoking status, and physical activity; ^∮^ Model 2 + intake of energy, vegetable, fruit, red meat, fish and green tea (or coffee); **^§^** Participants who had depression in the present or had ever depressed in the past year were regarded as having depression in the sensitivity analysis.

## Supplementary Materials

The following are available online at http://www.mdpi.com/2072-6643/10/9/1201/s1, Table S1: Characteristics of participants who were included in the analysis, and participants who were excluded from the analysis.
