# Supplementary material for: Green Tea, Coffee, and Caffeine Consumption Are Inversely Associated with Self-Report Lifetime Depression in the Korean Population
Source: Nutrients. 2018 Sep 1;10(9):1201. doi: 10.3390/nu10091201 (PMC6163318; doi:10.3390/nu10091201)
Supplement: Supplementary file 1 [file nutrients-10-01201-s001.pdf]

Supplemental Table S1. Characteristics of participants who were included in the analysis, and participants who were excluded from the analysis.

	**Included** (***n*** **= 9576)**	**Excluded** (***n*** **= 10023)**	***p*** **Value**
Age (year)	49.7 ± 0.3	51.4 ± 0.3	<0.0001
No. of participants (%)			<0.0001
Men	3852 (40.2)	4609 (46.0)	
Women	5724 (59.8)	5414 (54.0)	
Income level (%)			0.01
Low	2305 (24.1)	2440 (24.3)	
Medium	4896 (51.1)	4846 (48.3)	
High	2375 (24.8)	2737 (27.3)	
Educational level (%)			0.10
Elementary school (≤6 y)	2392 (25.0)	2214 (26.7)	
Middle/ high school (7–12 y)	4230 (44.2)	3657 (44.1)	
College or higher (>12 y)	2954 (30.8)	2413 (29.1)	
Smoking status (%)			<0.0001
Never	5810 (60.7)	4756 (47.5)	
Former	1901 (19.9)	1731 (17.3)	
Current	1865 (19.5)	3536 (35.3)	
Alcohol consumption (%)			<0.0001
Never	2584 (27.0)	2514 (30.6)	
< 2 times/week	5031 (52.5)	3965 (48.2)	
≥ 2 times/week	1961 (20.5)	1742 (21.2)	
Physical activity			
Regular	4518 (47.2)	3722 (37.6)	<0.0001
Body mass index (kg/m^2)	23.6 ± 0.0	23.7 ± 0.0	0.12
Energy intake (kcal/day)	2003.5 ± 10.8	1910.8 ± 12.7	<0.0001
Beverage intake			
Green tea (cups/week)	1.79 ± 0.05	1.97 ± 0.11	0.78
Coffee (cups/day)	1.33 ± 0.01	1.25 ± 0.02	<0.0001
Food intake (times/day)			
Vegetable	4.23 ± 0.03	4.25 ± 0.05	0.58
Fruit	1.05 ± 0.01	1.05 ± 0.02	0.60
Red meat	0.46 ± 0.01	0.44 ± 0.01	<0.0001
Fish	0.92 ± 0.01	0.93 ± 0.02	0.73

[a] Values are means ± SE or numbers (percentages); All *p* values are significant at $p < 0.05$; Data were missing for the following variables: education, $n = 1739$; alcohol consumption, $n = 1802$.
